# The Genomic Pattern of tDNA Operon Expression in *E. coli*

**DOI:** 10.1371/journal.pcbi.0010012

**Published:** 2005-06-24

**Authors:** David H Ardell, Leif A Kirsebom

**Affiliations:** Department of Cell and Molecular Biology, Biomedical Center, Uppsala University, Uppsala, Sweden; Washington University in St. Louis, St. Louis, Missouri, United States of America

## Abstract

In fast-growing microorganisms, a tRNA concentration profile enriched in major isoacceptors selects for the biased usage of cognate codons. This optimizes translational rate for the least mass invested in the translational apparatus. Such translational streamlining is thought to be growth-regulated, but its genetic basis is poorly understood. First, we found in reanalysis of the *E. coli* tRNA profile that the degree to which it is translationally streamlined is nearly invariant with growth rate. Then, using least squares multiple regression, we partitioned tRNA isoacceptor pools to predicted tDNA operons from the *E. coli* K12 genome. Co-expression of tDNAs in operons explains the tRNA profile significantly better than tDNA gene dosage alone. Also, operon expression increases significantly with proximity to the origin of replication, *oriC,* at all growth rates. Genome location explains about 15% of expression variation in a form, at a given growth rate, that is consistent with replication-dependent gene concentration effects. Yet the change in the tRNA profile with growth rate is less than would be expected from such effects. We estimated per-copy expression rates for all tDNA operons that were consistent with independent estimates for rDNA operons. We also found that tDNA operon location, and the location dependence of expression, were significantly different in the leading and lagging strands. The operonic organization and genomic location of tDNA operons are significant factors influencing their expression. Nonrandom patterns of location and strandedness shown by tDNA operons in *E. coli* suggest that their genomic architecture may be under selection to satisfy physiological demand for tRNA expression at high growth rates.

## Synopsis

The concentrations of tRNAs are co-adapted to codon usage frequencies in the transcriptomes of *E. coli* and other diverse organisms. But how are tRNA concentrations determined? Here, the researchers analyzed the *E. coli* tRNA concentration profile in its genomic context, using clustering and regression methods to partition tRNA concentration data to tDNA operons that were defined semi-automatically. They found that co-expression in operons explains the tRNA profile much better than tDNA gene dosage alone. Furthermore, they could significantly explain the total expression from tDNA operons by their distance from the genomic origin of replication. Per-copy transcription initiation rates from tDNA operons were also estimated. Although there is some evidence for replication-dependent effects on tDNA operon expression, this cannot explain how constant the tRNA profile is with growth rate. As a consequence, tDNA promoters are predicted to compensate for the location of their operons. Finally, the researchers found pronounced asymmetries between the leading and lagging genomic strands in the locations of tDNA operons, and on the effect of location on their expression. These nonrandom patterns suggest that the genomic location and strandedness of tDNA operons may be under some selection in *E. coli* to satisfy physiological demand for tRNAs at high growth rates.

## Introduction

During balanced growth in rich media, prokaryotic and eukaryotic microorganisms selected to grow efficiently are enriched in “major” isoacceptor tRNAs cognate to “preferred” codons in the transcriptome [1,2]. This is explained as a growth-maximizing strategy: to achieve a high rate of growth, ribosomes must be saturated with ternary complex (tRNA + elongation factor Tu + GTP) at the same time as mass invested in ternary complex must decrease [3]. From this perspective, ribosomal substrates specialize in major isoacceptors to optimize a trade-off between rate and mass of the translational apparatus [3]. We call this phenomenon translational streamlining.

There is a question as to whether the tRNA profile becomes increasingly enriched in major isoacceptors at higher growth rates. Some early studies of tRNA concentrations using Northern blots found this to be the case: major isoacceptors increased more than 4-fold at high growth rates, while most minor isoacceptor concentrations decreased [4,5]. A subsequent highly meticulous study using direct quantitation of radioactively labeled tRNA provides presumably the most precise, accurate, and complete measurements of tRNA concentrations in any organism to date [2]. Its authors find that concentrations of major isoacceptors increase with growth rate but only about 2-fold, from *μ* = 0.4 to *μ* = 2.5 doublings/h, less than had been found in the previous studies, while minor isoacceptor concentrations remained approximately the same. They conclude that the data are consistent with the hypothesis of growth-rate-dependent enrichment of the tRNA profile, a hypothesis that we call growth-regulated translational streamlining.

Although codon usage bias in efficiently growing microorganisms such as *Escherichia coli* has been considered one of the best examples of selection at the molecular level (see e.g., [6]), the factors that determine the cellular tRNA concentrations that co-vary with those codon usage patterns are still largely unknown. The mechanisms underlying growth-dependent modulation of tRNA concentrations have been called a mystery, and speculated to be elaborate. It is a de facto standard in computational studies to use tRNA gene (tDNA) dosage (i.e., copy number in the genome) as a proxy for tRNA concentration [7–9], yet in *E. coli,* gene dosage explains only about half of the variation in tRNA concentrations [2] at any growth rate. Gene dosage also cannot explain any eventual growth-rate-dependent modulation in the tRNA profile. tDNAs, like other genes, are organized into operons in prokaryotes, and it is natural to ask whether an operon-oriented perspective might afford a better understanding of the forces that determine the tRNA profile.

Furthermore, we wished to investigate whether the genomic organization of tDNA operons plays a role in determining the tRNA profile. Some tDNAs are found in common operons with ribosomal RNA genes (ribosomal DNAs, or rDNAs), and tDNA and rDNA operons have many upstream regulatory features in common [10]. There is a clear effect of genome position on the relative outputs of the seven *E. coli* rDNA operons: those closer to the origin of replication have relatively higher expression [11]. This is because in bacteria such as *E. coli,* which can divide faster than the time required for their genome to replicate completely, overlapping rounds of genome replication lead to a higher relative concentration of genes near the origin of replication [12]. The *dosage* of a gene is to be distinguished from its *concentration*. *Gene* *dosage* is the number of copies of a gene in a genome, and is static with respect to the physiological state of an organism or the replicative state of its chromosome. *Gene* (or *operon*) *concentration* is the average number per cell volume of a chromosomal region containing a gene or operon copy under specific “balanced” (that is, steady-state exponential) growth conditions. Different copies of the same gene scattered around in the genome will have different concentrations depending on the replicative state of the chromosome. Furthermore, gene concentrations depend on cell volume. Theory exists for calculating relative gene concentrations as a function of genome location, growth rate, and other physiological parameters [13–15]. This theory dictates that operon concentration increases exponentially with proximity to the origin of replication *(oriC)* at a given growth rate. Experimentally, transposition of certain reporter genes toward the origin of replication increases their total relative expression at a specific growth rate in a manner fully consistent with theory [14,16].

In light of these results, we wanted to ask whether replication-dependent effects of genomic location on operon concentration (position effects) can explain the biased tRNA profile in *E. coli*. Furthermore, we wanted to see if eventual growth-regulated translational streamlining is also mediated by position effects, if operons expressing major isoacceptors were seen to lie preferentially closer to *oriC*. We note in passing that, at least in *E. coli*, gene concentrations alone cannot explain the increased concentration of tRNAs at higher growth rates, since cell volume also increases exponentially with growth rate so that the concentration of *oriC* is kept approximately constant. This means that the concentration of all genes and operons everywhere else in the genome actually decreases with growth rate [14,15,17]. Therefore, the increasing concentration of tRNAs with growth rate [2] requires a growth-regulated increase in the output of all tDNA operons. We hoped then to describe this growth-dependent increase in the output of tDNA operons and see whether or not it was uniform.

However, other results speak against strong position effects explaining the tRNA profile or its eventual modulation with growth rate. The aforementioned connected problem of the regulation of ribosomal RNA (rRNA) synthesis has itself been the subject of controversy (reviewed in [18–20]). Alternatively to either gene concentration or dosage effects explaining variation, endogenous regulators likely induce feedback on stable RNA synthesis to maintain rRNA concentrations at systemically established levels. Different models have been proposed to explain, for instance, that ribosome concentrations are fairly stable to experimental alteration of rDNA operon dosage (reviewed in [18]). A further indication that operon concentration is not limiting to ribosome synthesis is that the synthesis rate is independent of cell age [21]. The effect of gene concentration on expression rate was specifically shown to be buffered in the case of another feedback-regulated system—namely, tryptophan synthase [14]. In the case of tRNAs, a recent study using microarrays also showed clear roles for processing and degradation on tRNA concentrations [22], suggesting that the idiosyncratic effects of individual tRNA structures and their precursors may have strong roles to play in explaining tRNA concentrations. Thus, it is far from clear that position effects can explain the tRNA profile either across operons within a given growth rate or across growth rates.

In the present work, we set out to re-examine Dong et al.'s data on the *E. coli* tRNA profile and its growth-rate variation in the genomic context of tDNA operon organization. We were surprised to find only weak evidence for growth-rate-dependent streamlining of the tRNA profile; instead, all tRNAs increase at very similar proportions and the tRNA profile is nearly equally streamlined toward major isoacceptors at all growth rates. Then we successfully mapped true tDNA operons in the *E. coli* genome using a simple, semi-automated scheme. With these in hand, we used least squares multiple regression and existing models and data for the physical properties of growing *E. coli* to estimate their total and per-copy expression. We show that this “operon model” explains the tRNA profile much better than gene dosage alone*.* We show that although a large fraction of the variation in tDNA operon expression must be explained by localized differences in regulatory elements and precursor structure, a significant fraction of variation in the *E. coli* tRNA profile is explained by the genomic location of tDNA operons. Our per-copy estimates, indicative of promoter strength, were consistent with independent experimental data and predict, surprisingly, that promoters in tDNA operons further away from *oriC* grow relatively stronger with growth rate. This may compensate for decreasing operon concentrations with growth rate to keep the tRNA profile constant. Finally, we demonstrate a significant asymmetry in the locations, and the effect of location on expression, of tDNA operons in the leading and lagging strands. Because co-expression in operons explains almost all of the variation in tRNA concentrations at any growth rate, and because tRNA concentrations are known to be co-adapted with codon usage, these results imply that the location and strandedness of tDNA operons may be partly influenced by natural selection in the genome of *E. coli*.

## Results/Discussion

### The tRNA Concentration Profile in *E. coli* Is Nearly Equally Streamlined at All Growth Rates

**Figure 1:**
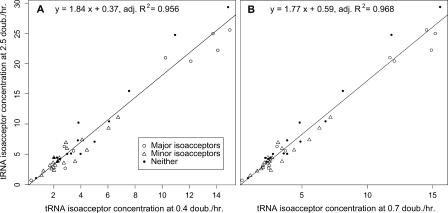
Regression of tRNA Isoacceptor Concentrations at the Highest Measured Growth Rate for *E. coli* Strain W1485 (A K12 Derivative) against the Same at Lower Growth Rates tRNA concentrations at the highest growth rate (*μ* = 2.5 doublings/h) are regressed against the same at (A) *μ* = 0.4 doublings/h and at (B) *μ* = 0.7 doublings/h. Concentration data are from [2]. Classification into “major,” “minor,” and “neither” types is from codon usage in ribosomal protein genes and anticodon reading relationships from [2,9]. All isoacceptors increase with growth rate, so that the uniform increase of all isoacceptors swamps variation in increase of individual isoacceptors.

In re-examining the data of Dong et al., we found that the concentration of all tRNA isoacceptors increases with growth rate, and does so with surprising proportionality. Figure 1 shows that linear regressions of tRNA isoacceptor concentrations at μ = 0.4 and μ = 0.7 doublings/h explain a surprisingly high fraction, upwards of 96% of the variation, in tRNA concentrations at 2.5 doublings/h. Both μ = 0.4 and μ = 0.7 are used because there may be some idiosyncratic aspects of the data at the lowest growth rates [23]. Thus, considering that the measurement error in the data of Dong et al. is 10%, the proportional increase of all isoacceptors with growth rate swamps any variation in the increase of individual isoacceptors.

**Figure 2:**
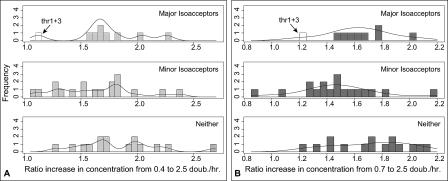
Frequency Histograms and Density Estimates for the Ratio Increase in Concentration of Different Classes of Isoacceptors After an Increase in Growth Rate Isoacceptors are grouped into “major,” “minor,” and “neither” classes, and the distributions of concentration ratios are shown for each class after an increase in cellular growth rate (A, light grey) from 0.4 to 2.5 doublings/h and (B, dark grey) from 0.7 to 2.5 doublings/h. White-colored bars correspond to values for Thr1+Thr3 as labeled (see text). While no difference is evident among classes from 0.4 to 2.5 doublings/h, a slight difference is evident from 0.7 to 2.5 doublings/h. This difference is not significant but becomes significant if Thr1+Thr3 and Pro1+Pro3 are removed from analysis, or trimmed means are used to compare groups.

Evidence for growth-regulated translational streamlining in the residual variation is weak. We classified isoacceptors as “major,” “minor,” or “neither” on the basis of whether they were cognate to preferred codons as described in the Materials and Methods section, and compared the distributions of ratio increases in concentrations of isoacceptors in these classes (concentration data and classifications are provided in Dataset S1). Figure 2 shows that the least increasing isoacceptors do fall in the minor class (containing 18 isoacceptors), while the major class (containing nine) shows a slightly greater increase with growth rate. Statistically, by this classification, the mean ratio increase of major isoacceptors is not significantly greater than that for minor isoacceptors. We used one-sided tests, which are liberal for rejecting the null hypothesis of equality of ratios between the major and minor groups. For the increase from 0.7 to 2.5 doublings/h (which shows the strongest difference), a Wilcoxon test finds a borderline difference between the distribution of major and minor isoacceptors (*p* = 0.06), a Welch's *t*-test on difference in mean ratios is also borderline significant (*p* = 0.08), but the bootstrap test on the difference in mean ratios is not significant (*p* = 0.10). Neither is an analysis of covariance test for the effect of isoacceptor type on concentration at μ = 2.5 controlling for concentration at μ = 0.7 (*p* = 0.37). *p*-Values for the increase from 0.4 to 2.5 doublings/h are all much higher, also failing to reject equality of means. Thus, the evidence for preferential enrichment of major isoacceptors with growth rate is not strong.

These results should be taken cautiously because they depend on how isoacceptors are classified. For instance, if we move Thr1+3 and Pro1+3 from the major class to the neither class, major isoacceptors do have a significantly higher mean increase with growth rate (Figure 2). Similarly, significance increases if we use trimmed means.

The Thr and Pro tRNAs belong to the only two isoacceptor families where major isoacceptors were not uniquely identified in our classification procedure (see Materials and Methods). Not coincidentally, these tRNAs are among the least abundant in the cell. This points out that it may not be correct to weigh all isoacceptor families equally in this analysis as we have done, because amino acid usage is biased and this bias increases with growth rate [24]. Furthermore, the classification is contingent on correct assignments of codon–anticodon reading pattern rules and preferred codons. Lastly, a more complete analysis could account for uncertainty in the concentration measurements. Nonetheless, it is clear that isoacceptor concentrations increase with growth rate in a much more proportional manner than was previously recognized. We conclude that there is scant evidence of growth-dependent streamlining of isoacceptor concentrations in favor of major isoacceptors.

Although our analysis is inconsistent with a strong effect of growth-regulated translational streamlining, it is not inconsistent with translational streamlining in general. Isoacceptor concentrations are biased in favor of major isoacceptors already at low growth rates. Even the slowest growth rate examined presents a significantly higher concentration of major isoacceptors by the Wilcoxon test (*p* < 0.01). This may be consistent with selection for translational streamlining at the highest growth rate determining the tRNA profile at all growth rates. In conclusion, growth regulation of the tRNA profile may be inessential to the theory that *E. coli* achieves a growth advantage through translational streamlining.

### Operons Explain tRNA Concentrations Better than Gene Dosage Alone

**Figure 3:**
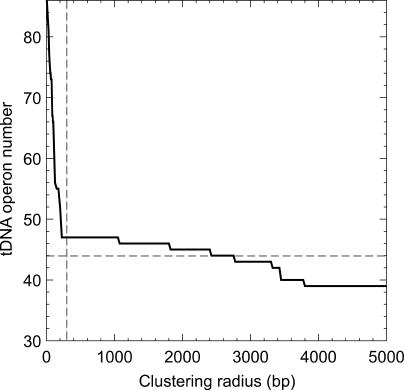
Effect of Clustering Radius on the Number of tDNA Clusters Obtained in the *E. coli* K12 Genome (Calculated in Steps of 25 bp) Clustering radius (*r*) is the maximum distance from one end of a tDNA in bp within which part of another co-linear tDNA must fall to be joined into the same cluster. Vertical dashed line shows the value used in this study (*r* = 300 bp), which correctly recovered all but three of the 44 experimentally known tDNA operons (indicated by horizontal dashed line). These three, ribosomal operons all, were not correctly recovered until a much higher radius was used but then within only a narrow range (2,400 ≤ *r* ≤ 2,800 bp) before a false positive was encountered. Thus, the natural proximity of tDNAs within operons made it possible through tDNA coordinates and strandedness alone to recover most of the true operons in *E. coli* K12.

Like protein-coding genes, tDNAs are co-transcribed in operons. We next set out to ask whether the operonic organization of tDNAs can better explain tRNA expression levels in *E. coli* at any growth rate better than gene dosage alone. We partitioned 87 tDNAs in the *E. coli* K12 genome [25] obtained with tRNAscan-SE [26] into 47 clusters. A tDNA or cluster of tDNAs was clustered together if they laid within 300 base pairs (bp) or less of one another (the clustering radius) and fell in the same strand. The clustering of 47 was stable for clustering radii between 200 and 1,000 bp (Figure 3). Although this procedure did split apart three rDNA operons—namely, *rrnC, rrnD,* and *rrnH*—known co-transcription relationships [10,27–30] were correctly identified in all other cases (including a previously unnamed operon containing only one Thr-2 tDNA, which we call *thrX;* for details, see Materials and Methods and [31]). We manually joined the three rDNA operons to produce a final set of 44 operons (Table 1).

**Table 1:**
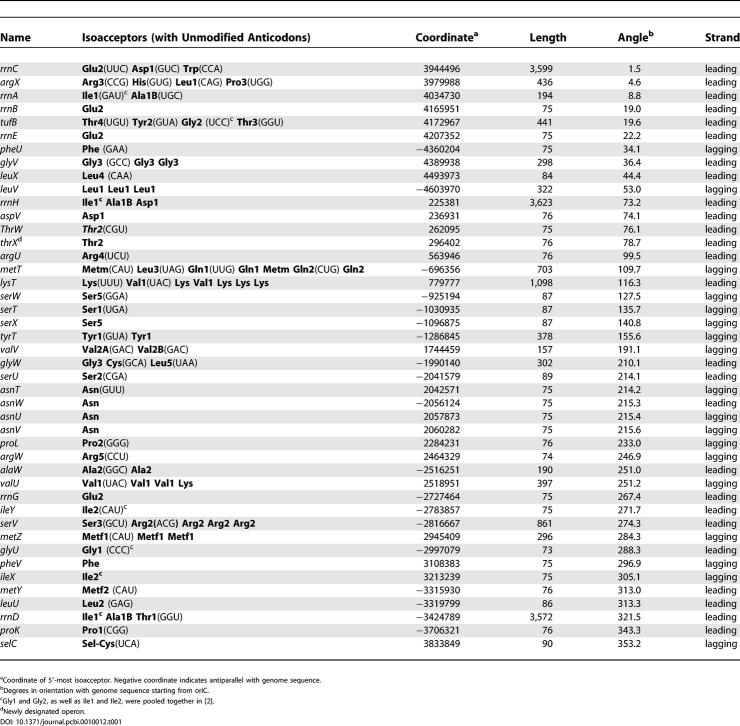
tDNA Operons in the *E. coli* K12 Genome

We used the primer sequences from [2] to map concentration data for 44 tRNA isoacceptors from each of five growth rates (Table 5 in [2]) to these tDNA operons, and then estimated by multiple linear regression by least squares with an intercept term, according to Equation 2 (Materials and Methods), a “standardized concentration” for each operon (the design matrix and corresponding right-hand side are provided in Datasets S2 and S3). We call this the operon model (with intercept) for explaining tRNA concentrations. Solving the operon model requires the placement of additional constraints on the system, as shown in Table 2 and described in Materials and Methods. For comparison to the operon model, we repeated the linear regression in [2] of tRNA concentration on tDNA dosage alone (“gene dosage model,” Equation 4 in Materials and Methods).

**Table 2:**
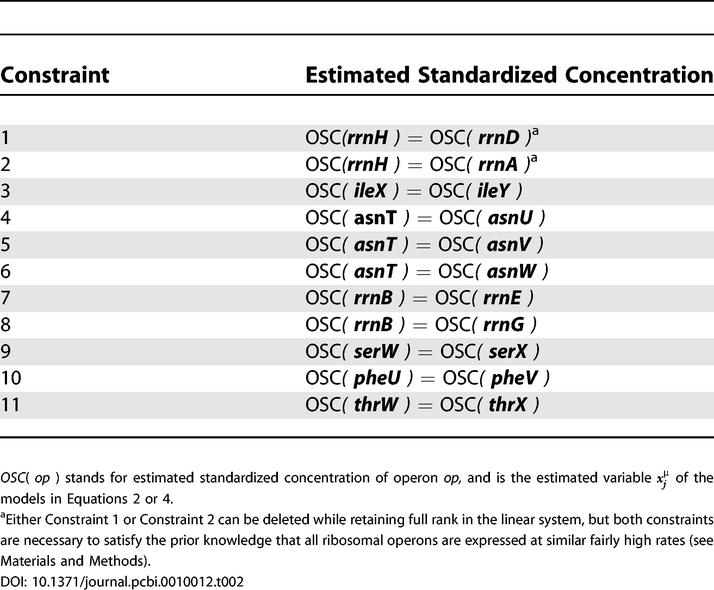
Constraints Added for Least Squares Estimation of OSCs

Despite the addition of 32 variables, the operon model explains tRNA concentrations statistically significantly better than the gene dosage model at all growth rates (*p*(*F*_32,10_) < 0.002 for all growth rates, see Table 3). Gene dosage explains only 55–60% of the variation, while the operon model explains 92–94% even after adjusting for the added variables (Table 3). This result suggests that, after controlling for gene dosage differences, inputs of different operons to the same isoacceptor pool, and the tendency for tDNAs to repeat within operons, tDNAs that are co-expressed in operons have significantly similar expression. We conclude that the operon model explains tRNA concentration data significantly better than gene dosage alone.

**Table 3:**
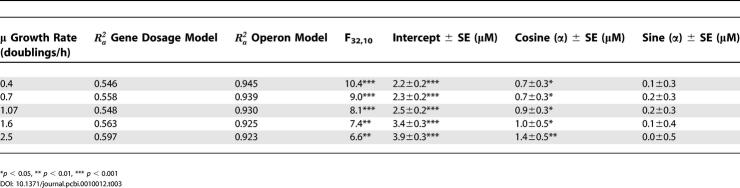
Comparison of Gene and Operon Models for Explaining tRNA Concentrations in *E. coli*, and Circular Regressions of the Estimated OSCs on Angle (α), Increasing as in min, but with the Origin of Replication at Zero in the *E. coli* K12 Genome

*p < 0.05, ** *p* < 0.01, *** *p* < 0.001

For subsequent work with the operon model, specifically in predicting operon expression, we redid the regression dropping out the intercept term, forcing regression through the origin. This is justified because both gene numbers and concentrations are on ratio scales and concentrations have a natural zero if the number of their encoding genes are zero. That is to say, the intercept term has no clear biological interpretation. On the other hand, for model comparisons and regression statistics just shown, we retained intercepts both to reproduce earlier work and because doing so is recommended statistical practice [32]. Intercept terms were never significant in our regressions. At low growth rates (0.4, 0.7, and 1.07 doublings/h), intercept terms were about 70% of the mean magnitude of coefficient estimates, and could reduce their value by half. At high growth rates, intercept terms were about 5% of the mean magnitude of coefficient estimates and affected coefficient estimates by only about 10%.

**Figure 4:**
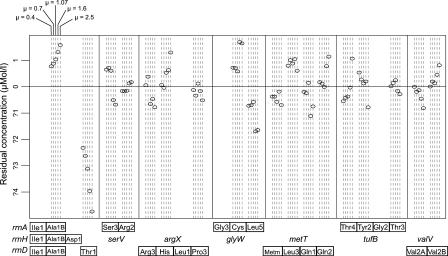
Residuals of the Operon Model Regressions Used to Estimate Expression (Without Intercept), Showing Unexplained Variation Only residuals with absolute values larger than 10^−14^ are shown. Thus, variation in all but nine of the 44 operons is completely explained. All operons containing tDNAs with these non-zero residuals are arrayed at the bottom, showing true tDNA order from 5′ to 3′. All such tDNAs are in single-copy except for the genes encoding Ala1B in the ribosomal operons, indicated by vertical stacking at the bottom, Arg3*,* which is repeated three times in the *serV* operon, and MetM*,* Gln1*,* and Gln2*,* which are each repeated twice in the *metT* operon (for its true configuration, see Table 1). Residuals are shown in μM units, those of standardized concentrations. Residuals are shown for each tDNA in order of increasing growth rate from 0.4 (leftmost) to 2.5 doublings/h (rightmost). Positive (negative) residuals indicate underestimation (overestimation) by the model.

In the operon model, there are no polarity effects, so that contributions of gene copies are independent of location within an operon. We found that the improvement of the operon model over the gene dosage model (with or without intercept, data not shown) increases quite a bit if we do not manually join the proximal and distal tRNA genes from the three previously mentioned rDNA operons: without intercept, the probability of fit improvement by chance decreases by two orders of magnitude (*p*(*F*_33,10_) < 10^−5^), and the adjusted fraction of variation explained is greater than 98% at all growth rates (design matrix and right-hand side provided in Datasets S4 and S5). This model improvement from not joining the distal tDNAs of the ribosomal operons may be because of degradation, RNA polymerase drop-off, or decoupling through secondary promoters, yielding the most influence on the rDNA operons because they are the longest among our operon set. Indeed, initial analysis of promoters in our tDNA operons indicates that the distal *Thr1*-tDNA in the *rrnD* operon may have a secondary promoter while the distal tDNAs in the *rrnC* and *rrnH* operons do not [31]. This is borne out by an examination of residuals from the operon model without intercept (Figure 4). However, the residuals do not show a generally consistent trend of overestimated expression from operon distal ends, which would have been consistent with systematic transcriptional drop-off (or other effects of operon polarity) on tRNA concentrations.

### tRNA Operons Are More Productive the Closer They Lie to *oriC*

We then re-estimated operon standardized concentrations (OSCs) by repeating the operon model regression dropping the intercept term. OSCs (the 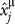 obtained by fitting the model in Equation 5) estimate, for each operon and growth rate, the concentration that a tRNA isoacceptor would have at that growth rate if its gene were contained in single copy in, and only in, that operon. Alternatively, OSCs estimate the hypothetical concentrations of tRNA precursors that would be expressed from each operon in the absence of tRNA precursor processing, all other factors being equal. Estimated OSCs and other data about operons are provided in Dataset S6.

In balanced growth, tRNA concentrations, like the concentrations of all cellular components, are proportional to their rates of synthesis (see e.g., [18]). Assuming that tRNA precursor processing is fast and the degradation of stable RNA is slow, this means that tRNA concentrations in balanced growth are proportional to their rates of transcription. Therefore, under these assumptions, our estimated OSCs at a given growth rate are proportional to the “bulk” rate of transcription from each operon at that growth rate, with different constants of proportionality at different growth rates. In the Materials and Methods section, we show how to calculate these constants of proportionality. Below, we present some statistical analyses in terms of OSCs, but equivalently refer to them in terms of operon bulk expression rates when, and only when, such results are invariant up to a multiplicative constant that is equivalent in statistical analysis.

**Figure 5:**
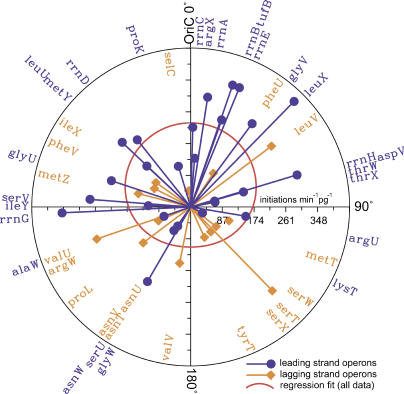
Total Expression (Bulk Output) at *μ* = 2.5 Doublings/h of tDNA Operons Against Their Location in the *E. coli* K12 Genome The angular scale is symbolized by α in the text, with 0° placed at the origin of replication *(oriC)* shown at the top. Units of the radial axis (expression) are initiations per min per picogram of culture mass. Leading strand operons are indicated in blue and lagging strand operons in gold. The red curve shows a re-estimated circular regression of all the data including only intercept and cosine terms, showing the significant tendency of expression to increase toward *oriC,* especially for leading strand operons. Values for lagging strand operons *asnV, asnU,* and* asnT* are covered but equal by constraint (see Table 2) to the value for *asnW*.

We explored the spatial variation in the genome of operon expression by plotting OSCs against the genomic location of operons and through the use of circular regressions [32], where 0° was placed at the origin of replication *oriC*. Including an intercept term in a circular regression of OSC (or, equivalently, bulk expression) on genome location (see Equation 6), we found significant negative dependence of operon bulk expression on distance from *oriC* at all five growth rates, as indicated by the significant cosine terms in Table 3. In contrast, sine terms of the circular regressions were not significantly different from zero, suggesting symmetry of the expression pattern about *oriC.* Figure 5 shows that, despite considerable variation independent of location, average bulk expression of operons (for μ = 2.5 doublings/h in units of initiations per minute per pg of cell culture) increases the closer that operon lies to *oriC*. Figure 5 also shows a re-estimated circular regression including only the cosine and intercept terms, which we later use to compare models in Table 4.

**Table 4:**
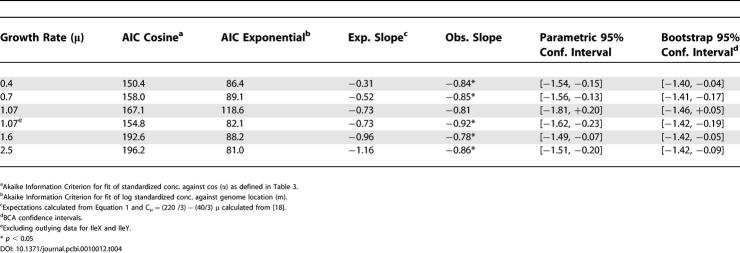
Evidence for Log-Linear Fit of Estimated OSCs (μM) against Genome Location (*m*)

^a^Akaike Information Criterion for fit of standardized conc. against cos (α) as defined in Table 3.

^b^Akaike Information Criterion for fit of log standardized conc. against genome location (m).

^c^Expectations calculated from Equation 1 and C_μ_ = (220 /3) − (40/3) μ calculated from [18].

^d^BCA confidence intervals.

^e^Excluding outlying data for IleX and IleY.

* *p* < 0.05

Proximity to the origin of replication explains 11–17% of the variation in estimated tDNA operon expression in *E. coli* in a circular regression. However, it is not at all clear whether this implies that expression rate is a cause or an effect of location in the genome, or both. Expression rate as an effect of location could derive either physiologically, by the effect of genome position on gene concentration, or evolutionarily, by some (admittedly highly speculative) genome-position-dependent mutation effect that would tend to make promoters stronger near the origin of replication. Expression rate as an ultimate cause of location could occur through the selection of certain operons to be retained near *oriC* after they are moved there by translocation, horizontal transfer, or inversion of operons and tDNAs in the genome. This selection could be for higher expression through the position effect on expression or by some hypothetical advantage of operons with strong promoters per se to lie near the origin. In the following, we find evidence of both directions of causality: expression is caused to some degree by genome location, but the location and strandedness of operons has also likely evolved in *E. coli* to exploit this and other effects to increase satisfaction for tRNA demands of the cell.

### tRNA Operon Expression Is Consistent with Replication-Dependent Gene Dosage Effects

To examine the hypothesis that position-dependent effects of replication on gene concentration are causing the genomic pattern of tDNA operon expression in *E. coli*, we compared our estimated operon expressions to a statistical model based on well-known theory relating gene expression to gene concentration [14,15]. The full derivation of this model is shown in Materials and Methods. This model is as follows: 


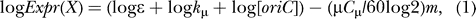


 where *Expr(X)* is the expression of operon *X* at growth rate *μ* and genomic position *m* (as a fraction of the length of half of a chromosome), *k_μ_* is an unknown proportionality constant relating the average expression of a set of genes to their concentrations, *ɛ* is a stochastic error term, *[oriC]* is the concentration of the origin of replication, and *C_μ_* is the time required for complete genome replication, considered a constant given the bacterial strain and growth rate *μ*.

The intercept in this model depends on unknown factors or nuisance parameters such as the concentration of *oriC* in relation to growth rate (which can vary at low growth rates and is possibly strain-dependent [18,33,34]), the distribution of the stochastic error *ɛ*, and *k_μ_,* which captures the uniform increase in transcription at all tDNA operons as a function of growth rate. However, the slope in this model, and its underlying exponential form, depends on only the well-studied parameter *C_μ_,* and can be directly compared to our expression estimates to evaluate consistency with position effects on gene concentration.

We find general agreement of our estimates with the model in Equation 1. That is to say, we find evidence for exponential fits of operon expression against genome location, decreasing from the origin. First, the Akaike Information Criterion indicates that an exponential model (Equation 9) fits the standardized concentration data much better than the trigonometric model (Equation 6) used in the circular regressions (Table 4). This means that the data are more consistent with an exponential trend than with the trigonometric trend implied in the circular regression, although it does not rule out that some other function would fit the data better. Second, the Box-Cox test suggests log-transformation of the data, also consistent with an exponential trend (Figure S1). Indeed, the 95% confidence intervals of lambda at all growth rates include zero (log-transformation) and exclude one (no transformation of the data). Third, confidence intervals for the slopes in log-linear fits include expected values based on realistic estimates of *C* at all growth rates (Table 4). Thus the data are broadly consistent with a position effect on gene concentration causing increased expression of operons toward *oriC*.

On the other hand, for the model in Equation 1 to fit in detail, one would expect the slopes of the log-linear regressions in Table 4 to decrease with increasing growth rates. One expects, in other words, steeper gradients of expression on distance from the origin of replication at higher growth rates. However, our estimated slopes are constant in *μ*. Indeed, operon expression rates, like raw tRNA isoacceptor concentrations, increase in fairly constant proportions with growth rate. High fractions, albeit lower than with the raw data, of estimated operon expression rates at higher growth rates could be explained by those at lower growth rates, from 82–97%. For instance, when regressed through the origin, the growth-dependent increase factor at 2.5 doublings/h from 0.4 doublings/h is 1.74 ± 0.04 (SE) for tDNA estimated operon expression rates and 1.80 ± 0.04 for raw tRNA concentrations, explaining 97% and 98% of the variation, respectively. This shows that the data are inconsistent with gene concentration as a cause of growth-regulated translational streamlining.

It is remarkable that if *E. coli* actually were selected for extensive growth-regulated translational streamlining, this could have easily been arranged by holding the relative strength of different tDNA promoters constant with growth rate and passively exploiting the location effect on gene concentration. The fact that the tDNA operon expression profile is fairly constant, despite changes in underlying operon concentrations, implies that the relative strength of different tDNA promoters must change with growth rate. We can ask which growth rate condition better fits the expected slope, which might suggest under which condition expression is most governed by the position effect on operon concentration and least governed by a hypothetical compensating factor working against this effect. Interestingly, the data fit the expected slopes better at higher growth rates (Table 4), suggesting that any hypothetical compensatory effect may be most effective under slow growth conditions. In this case, this hypothetical compensating effect would make, at low growth rates, either origin-proximal promoters stronger, terminus-proximal promoters weaker, or both, than what would be expected based on comparisons with expressions at higher growth rates.

We can also regress out the expected position effect on operon concentration to study trends in estimated per-copy expression rates of each tDNA operon at each growth rate, using the derivation shown in Materials and Methods (Table 5). Our data agree fairly well with independent calculations for rDNA *(rrn)* operons [35]. According to these calculations, the average expression from all *rrn* operons combined increases from 8.9 initiations per min per copy at μ = 0.7 to 16.0 at μ = 1.07 and up to 66 initiations per minute per copy at μ = 2.5 doublings/h (Table 5). These estimates are quite similar, if slightly under, those estimated in [35] based on different assumptions, data, and bacterial strains. Based on measurements of total RNA, they calculated the average initiation rate of *rrn* operons to be about 10 at μ = 0.6, just under 20 at μ = 1.1, about 64 at μ = 2.2, and almost 90 at μ = 2.7. The data in [35] are presented only as figures, not tables, so the values quoted here are approximate. The agreement seems satisfactory. Our estimates for *rrn* operons may be low because their distal tDNAs are weighted equally (see above), and because of the relative lack of information for the isoacceptors they express (i.e., Ile-tRNAs). Nonetheless, these results suggest that Table 5 shows reasonable predictions for initiation rates from tDNA operons, with one caveat: excess synthesis to compensate for eventual tRNA degradation, or for transcriptional abortion and drop-off, cannot be detected from the combination of data and theory we have used to make these estimates. The estimates in Table 5 should therefore be considered as minimal estimates assuming no losses, or as “net” synthesis rates after such unmeasured losses have taken place.

**Table 5:**
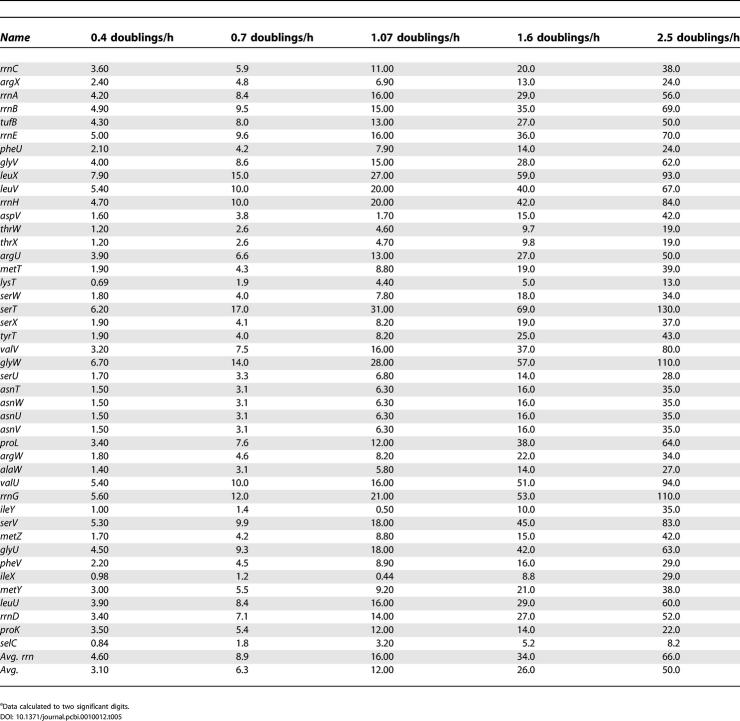
Estimated Average Synthesis Rates Per Operon (Number of Transcripts Initiated per min per Copy) of tRNA Precursors in the *E. coli* K12 Genome^a^

^a^Data calculated to two significant digits.

**Figure 6:**
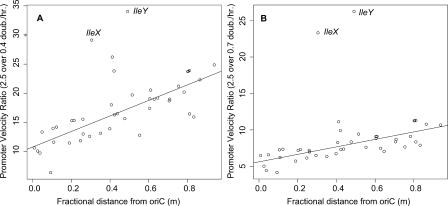
Ratio Increase in Per-Copy Synthesis Rates of Operons (Promoter Velocities) as Growth Rate Increases from *μ* = 0.4 to 2.5 Doublings/h (A) μ = 0.7 to 2.5 doublings/h. (B) Against fractional distance from the origin of replication *oriC* with maximum distance set at 1 *(m).* Outlying values for *ileX* and *ileY* are indicated.

The per-copy estimates of operon expression rates (“promoter velocities”; 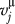 in Materials and Methods) have no significant trend against genome location at any growth rate. This argues against strong promoters per se evolving near *oriC* either by location-dependent mutation effects in situ or by translocation. However, the growth-rate ratio increase in per-copy synthesis rates are very significantly and positively dependent on distance from *oriC* (Figure 6), both from *μ* = 0.4 to *μ* = 2.5 ( 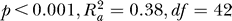 ) and *μ* = 0.7 to *μ* = 2.5 ( 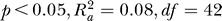 ). Significance and explained variation increases when we exclude outlying values for the *IleX* and *IleY* operons ( 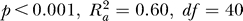 and 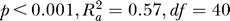 **,** respectively). This is the evidence for the aforementioned compensatory effect holding the tRNA profile relatively constant despite the position effect on operon concentration. Since we have not examined any model for the growth regulation of tDNA promoters as a whole, we cannot say whether this compensatory effect acts through greater acceleration of origin-distal promoters or lesser acceleration of origin-proximal promoters at higher growth rates.

To summarize, we have provided strong evidence that the genomic location of tDNA operons plays a significant role in shaping the tRNA profile in *E. coli*. Even though it is obvious that location-dependent gene concentrations must be accounted for when calculating expression rates from any one operon, our result that such position effects partly explain concentration variation across different tRNAs is unexpected and novel. Yet the results are not fully consistent with the simplest model of operon location determining expression rate. First, location explains only about 15% of expression rate variation (Figure 5), so one may say that intrinsic causes of expression such as promoter velocity are the primary determinant. Second, the tRNA profile is relatively constant at different growth rates while tDNA operon expression is a negative exponential function of the product of growth rate and genomic distance from *oriC* (Equation 1). This implies the existence of a compensatory effect working against the position effect on expression (Figure 6).

### tRNA Operon Locations and Expression Are Different in the Leading and Lagging Strands

tDNA operons in *E. coli* are different in the leading and lagging strands with respect to both location and the effect of location on expression. We defined the strandedness of an operon by the angular coordinate relative to *oriC* (α) of its first tDNA and its orientation relative to the K12 genome sequence; if *α* < 180°, the operon is leading if parallel and lagging if antiparallel, and vice versa otherwise (see Table 1). The leading and lagging strands are quite different with respect to the placement and the expression of tDNA operons (see Figure 4). tRNA genes considered separately lie preferentially on the leading strand (*χ* ^2^ = 5.069, *df* = 1, *p* = 0.02), but this ignores operonic organization with its constraints of co-orientation and tendency for tDNA repeats within operons. We find that tDNA operons are evenly distributed on the two strands (*χ* ^2^ = 1.454, *df* = 1, *p* = 0.23). Furthermore, statistically speaking, there is no difference in the mean size (number of tDNAs) of leading and lagging tDNA operons, by either a permutation test (*p* = 0.35; see Materials and Methods) or the Wilcoxon test (*p* = 0.52). However, after dividing operons into two sets according to whether they lie closer to *oriC* or the terminus region, leading strand operons lie significantly closer to the origin and lagging strand operons closer to the termini (Figure 5 and Fisher's exact test, *p* = 0.010). This observation is partially reaffirmed by circular statistics [32,36]. The Watson two-sample test rejects homogeneity of the spatial distributions of leading and lagging strand tDNA operons (U^2^ = 0.1949, *p* < 0.05) and the Rayleigh test rejects circular uniformity of the placement of leading strand operons against an alternative unimodal distribution toward the origin (*r*_0_ = 0.3214, *p* < 0.01).

However, the distribution of lagging strand operons is not significantly different from uniform by any circular statistical one-sample test that we tried, including Watson's, Kuiper's, and Rayleigh's. Although these tests make different assumptions, they all lead to the same conclusion. Lack of power without including prior knowledge of the biological importance of the origin or termini may partly explain this, as these tests also failed to reject uniformity for leading strand operons. When we supplied an alternative hypothesis that lagging strand operons are oriented toward the termini, the Rayleigh test for lagging strand operons barely failed to reject uniformity (*r*_0_ = 0.2446, *p* = 0.07). We conclude that leading strand tDNA operons are significantly clustered spatially toward *oriC* while lagging strand operons are not.

With respect to expression, leading strand operons show an even greater increase in estimated expression toward *oriC* than all operons taken together (cosine term 2.07 ± 0.75, *p* = 0.0108, sine term NS), while lagging strand operons alone show no significant relationship of expression on genome location. However, by either analysis of covariance or a Wilcoxon test of residuals, we found no effect of strand—that is, no statistical difference between the two strands—on estimated bulk expression of operons at any growth rate, after accounting for the effect of operon location.

Thus, in *E. coli,* genome location effects are very strong in the leading strand and statistically insignificant in the lagging strand. In the leading strand, operons are placed nonrandomly with respect to *oriC,* and there is a strong location effect on expression. However, operons and overall expression are both equally distributed on the two strands. How do we explain these statistical observations? Rocha discusses two hypotheses to explain strand asymmetries in gene placement and gene expression [37]. Both depend on the effects of head-on collisions of DNA polymerase and RNA polymerase during the transcription of lagging strand genes. One hypothesis emphasizes the effect of such collisions on genome replication through stalling of replication forks. The other hypothesis emphasizes the effect of such collisions on transcription through aborted transcripts either failing to meet gene product demand or by their dominant negative effects due to hypothetical toxicity. We speculate that transcriptional output might be diminished after a head-on collision of RNA polymerase with the replication fork not only by abortion of an elongating transcript at the time of collision but also possibly by interference with transcription during resolution of stalled replication forks after a collision.

Systematic studies of protein-coding genes in *E. coli* and other bacteria have pointed toward gene essentiality rather than gene expression level as a better explanatory variable for predicting strandedness [38]. This favors the interpretation that it is the effect of polymerase collisions on transcription that determines strand asymmetries in gene placement. With tRNA precursors, it seems unlikely to us that incomplete transcripts would have toxic effects, but a detrimental effect from failing to meet the physiological demand of translation seems likely. Constraints of high demand on an operon might select on it being located near the origin with leading strand orientation, while lesser demand might permit an operon to evolve a more random location and orientation through transposition and inversion, with adjustments by the local evolution of promoter strength as the dominating factor setting expression levels in both strands. We note in passing that if there were a toxic effect from the transcriptional abortion of any operon through fork collision with RNA polymerase, it would be amplified by genomic proximity of offending operons to the origin of replication, since the toxicity would be proportional to average gene concentration (the proportion of toxic product to total production from an operon would be independent of location, but the absolute quantity of toxic product would increase with the concentration of the operon). We speculate further that the negative effect of lagging-strandedness on transcriptional productivity would be a constant proportion of output regardless of location, and might therefore be expected to be neutral to location. These speculations demand a quantitative assessment of the data on the mechanisms and kinetics of events during and after such collisions for their further evaluation.

Our results lead us toward the testable conclusion that physiological demand for tRNA can serve as an evolutionary cause of the genomic location and strandedness of tDNA operons. The tRNA profile, which is known to be selected for translational streamlining in covariation with preferred codons, has now been shown to be correlated with the location and strandedness of tDNA operons. This suggests that the genomic architecture of tDNA operons is itself under some degree of natural selection in *E. coli*.

Stronger conclusions from the present analysis cannot be made before comparative analysis of tDNA operon and promoter sequences is undertaken within and among enterobacterial genomes. This will be the subject of future research.

## Materials and Methods

87 tDNAs were identified by tRNAscan-SE [26] from the *E. coli* K12 MG1655 genome and then matched by regular expressions to reverse complements of oligo probe data from [2]. Only one tDNA was unmatched by any of the oligos: one of the major Leu1 tDNAs with a single mismatch in the variable loop from other copies. This mismatch occurs in the middle of the relevant oligo, so this tDNA was included. We included a Thr2 gene we call *thrX* at coordinate 296402. This Thr2 gene is annotated in the Genome tRNA Database (http://lowelab.ucsc.edu/GtRNAdb/), but it is not annotated in EcoCyc (http://www.ecocyc.org) nor in the NCBI genome annotation, and there is no evidence for its specific expression. Using tRNAscan-SE, the *thrX* gene has a covariance score of 42.36, which is almost half that of the other and nearby Thr2 gene *thrW* at coordinate 262095, indicating that it contains structural irregularities. Closer inspection shows that 1) *thrX* is a chimera with a 3′ end identical to *thrW* starting in the anticodon stem 5′ of the loop, 2) that the oligo used against Thr2 in [2] matches the 3′ end of the anticodon stem into the variable loop and would therefore hybridize perfectly to this tRNA were it expressed, 3) this tRNA would probably fold normally if it were expressed, including tertiary contacts, and 4) the *thrX* gene has a reasonable upstream promoter [31]. We therefore included *thrX* in the analysis, but removing it from analysis does not change our regression results (design matrix and right-hand side provided in Datasets S7 and S8), because the only other operon containing a Thr2 gene in *E. coli* K12 is nearby and co-oriented *thrW*.

All statistical analysis was executed in R [39]. We classified isoacceptors as “major,” “minor,” or “neither” in reference to preferred codons in highly expressed genes at high growth rates. For this codon-based criteria, we analyzed codon usage in 45 ribosomal protein genes from the *E. coli* K12 genome [25] with codonw (J. Peden, http://www.molbiol.ox.ac.uk/cu/). The top two codons for each amino acid were checked against cognate anticodon reading patterns as according to [2], which is also the source of the correspondence between tRNA isoacceptor numbering and anticodons shown in Table 1. A major isoacceptor was then picked uniquely in all acceptor classes except for two cases (threonine with two tRNAs, Thr1 and Thr3, that both have GGU anticodons matching the preferred codons ACC and ACU, and proline with two tRNAs, Pro1 and Pro3, that both match the preferred codon CGG). Thr1 and Thr3 were added together as were Pro1 and Pro3, and these combined data were assigned to the major class. This assignment of major isoacceptors is identical to that used by Ikemura [40] with the exception of Ser5, which Ikemura did not measure. Two undecidable cases (Ile1+Ile2, which Dong et al. could not distinguish, and Tyr1 and Tyr2, both of which match both Tyr codons) and all tRNAs in single-isoacceptor families were labeled as “neither.” The remainder were assigned to the minor isoacceptor class. The classifications are shown in Dataset S1. The bootstrap test for difference in mean ratios between the major and minor groups was calculated by algorithm 16.2 on p. 224 in [41]).

To predict operons, tDNAs with the same orientation in the genome were clustered automatically using an end-to-end distance (clustering radius) of 300 bp. In known cases of heterogeneous operons (tDNAs mixed with protein-coding genes), tDNAs always come first in the operon [28]. We have found no evidence otherwise in a separate analysis of upstream promoters [31]. The procedure split apart three ribosomal operons that were manually joined as described in the text.

OSCs are derived from concentration data in Table 5 in [2] projected onto the defined operons by least squares multiple regression and are in μM units (see Dataset S5). In order to perform this operon model regression, we had to add ten additional constraints first to bring the design matrix to full rank, and then one additional constraint to enforce nearly equal expression of ribosomal operons (see Table 2). Ten of the 11 constraints amounted to adding assumptions, such as if there are two operons feeding into, and only into, a single isoacceptor pool, that they do so equally and were absolutely necessary to perform the regression. One of these ten involved constraining two of the three ribosomal operons *rrnA, rrnD,* and *rrnH* and was necessary to perform the regression, but involved a choice between two alternatives. However, with either of these minimal ten constraints, the two constrained *rrn* operons were estimated to be expressed at unrealistically low levels and the other at an unrealistically high level, when it is known that all ribosomal operons tend to be expressed at similar fairly high levels [10,11,42] (Design Matrix and right-hand side provided in Datasets S9 and S10). This may have been because there is relatively little data for the isoacceptor pools fed into by ribosomal operons—for instance, Dong et al. were not able to distinguish the Ile1 and Ile2 isoacceptors. Therefore, we added back the additional constraint on these ribosomal operons to enforce their nearly equal expression (see Table 2).

We estimated OSCs 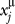 of operon *j* at growth rate *μ* by least squares solutions of matrix equations of the form


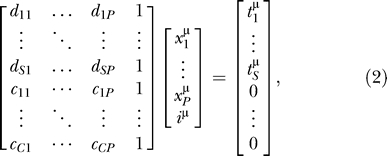


where *S* is the number of isoacceptors, *P* is the number of operons, *C* is the number of constraints, 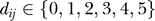 is the dosage of tDNA *i* in operon *j,* 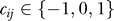 is the coefficient of constraint *i* on operon *j,* *i^μ^* is an intercept term, and 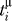 is the concentration of tRNA *i* at growth rate *μ*. For ease of reference, we can rewrite Equation 2 as


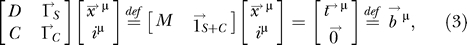


where *D* is a *S* × *P* matrix, *C* is a *C* × *P* matrix, *M* is a (*S* + *C*) × *P* matrix, 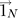 is a column vector of ones of length *N,* 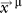 is a column vector of 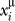 of length *P,* 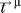 is a column vector of 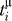 of length *S,* 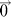 is a column vector of zeroes of length *C,* and 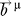 is the concatenation of 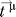 and 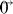 .

We call a specific matrix *M* on the left-hand side of Equation 3 a “design matrix.” For what we call the operon model, *S =* 44,* P =* 44, and *C =* 11. All design matrices *M* and their corresponding 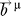 are available in Datasets S2–S5 and S7–S10. For comparison to the operon model, we repeated the linear regression in [2] of tRNA concentration on tDNA dosage alone (gene dosage model), which assumes equal expression of all tDNAs, thereby fitting concentration data using only a single variable *x^μ^* and genomic tDNA copy number as a predictor. In our notation, the gene dosage model is simply


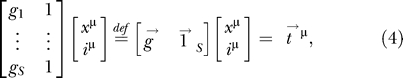


where 
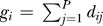
 , and 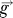 is a column vector of size *S,* the *i^th^* component of which is *g_i_*.

For the purposes of evaluating and comparing the operon and gene dosage models ( 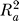 and *F* in Table 3), we included intercept terms in the regressions. The degrees of freedom are 42 for the gene dosage model and 10 for the operon model (44 data points minus 44 operons plus 11 constraints minus 1 intercept term). The adjusted coefficients of determination ( 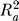 in Table 3) are corrected for degrees of freedom by the definition 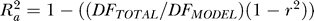 .

For the purpose of studying genomic variation in operon expression under the operon model, we regressed the data without an intercept term according to the model


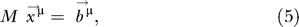


which reasonably implies that these operons are the only sources of tRNAs in the cell, and is also reasonable because tRNA concentrations and gene dosage are on ratio scales with true zeroes.

The circular regression presented in Table 3 uses a model


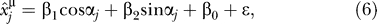


where 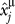 is the standardized concentration of operon *j* estimated according to the model in Equation 5, *α_j_* is angular distance of operon *j* from *oriC* in the direction of minutes of genetic distance, *β_I_* are regression coefficients, and *ɛ* is an error term. The regression shown in Figure 5, and the cosine model in Table 4, is based on this model without the sine term, which was insignificant at all growth rates. In the case of Figure 5, the fitted model was scaled by a constant to give predicted bulk operon expressions. The calculation of this constant is described next.

Bulk operon expression (total expression rates) are observed (estimated by fitting the model in Equation 4) or predicted (from a fit to the model in Equation 6) OSCs 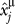 multiplied by a growth-rate-dependent factor *r_μ_* = (*N_A_V_μ_*/10^21^ *M_μ_*)(*μ*ln2/60), where *N_A_* is Avogadro's number, *μ* is the growth rate in doublings/h, *V_μ_* is average cell volume in μm^3^, and *M_μ_* is average cell mass in grams as functions of *μ*. Multiplication by this factor yields units of number of initiations per min per gram of cell culture. Functional relationships for average cell volume (*V_μ_* = 0.4 × 2*^μ^* μm^3^) and average cell mass (*M_μ_* = 1.6 × 10^−13^ × 2*^μ^* g) with growth rate *μ* were taken from [17]. The first factor in *r_μ_* yields a density while the second factor in *r_μ_* derives from the relationship between density and synthesis rate during balanced growth [18]. Values in Figure 5 are multiplied by an additional factor of 10^−12^ to yield values per picogram. Statistical results calculated on data within a growth rate (such as in Tables 3 and 4) are invariant to multiplication by this constant factor. Therefore, some results are discussed and presented equivalently as standardized concentrations or total expression rates.

The average concentration *[X]* of a gene per concentration *[oriC]* of the origin of replication *oriC* at growth rate *μ* and location *m* (relative distance from the origin of replication as a fraction of maximal distance, with the length of a half-chromosome set to 1) follows the relationship [14,15]:


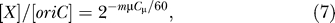


where *C_μ_* is the time required for complete genome replication, considered a constant given the bacterial strain and growth rate *μ* (equivalently, this formula can be presented in terms of the doubling time in min *τ,* where *τ = 60/μ*). A derivative stochastic model for the predicted expression *Expr(X)* of such a gene is therefore


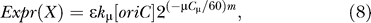


where the unknown proportionality constant *k_μ_* relates the estimated average expression of a set of genes to their concentrations as an unknown but common function of growth rate, and *ɛ* is some stochastic error term. Taking logarithms yields Equation 1 in the text.

The Box-Cox tests the likelihood of different functional families with the data using a single parameter lambda. We used the default range in R to fit lambda, which is from −2 to 2 in 0.1-increments.

Table 4 compares the circular regression model in Equation 6, fitted without a sine term, to the fit of the data to an exponential model of the form in Equation 1, namely:


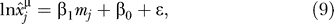


where *m_j_* is the fractional distance from *oriC* of operon *j* with maximum 1. The expected slopes in Table 3 are calculated from Equation 1 and the assumption of a linear and strain-independent [33,34] dependence of the genome replication period *C* on growth rate *μ,* calibrated from data in [18,33] to be *C_μ_* = (220/3) − (40/3)*μ*. We also repeated these comparisons excluding outlying estimates for the *ileX* and *IleY* operons. That these estimates were outliers could be seen by comparing relative proportional trends of estimates against growth rates (e.g., Table 5 or Figure 6), as well as by their effects on statistical tests (e.g., Table 4). Instability in these estimates came because of the aforementioned lack of data for Ile isoacceptors (Ile1 and Ile2 could not be distinguished by the oligos in Dong et al.'s data) relative to their constraint in the least squares regression.

Per-copy estimates of operon expression rates, which we also call “promoter velocities” 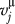 (see text for caveats) and shown in Table 5, are proportional to bulk operon expression rates 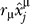 through an additional growth-rate-dependent factor 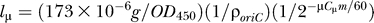 . The first factor in *l_μ_* converts grams to spectrophotometric units *OD_450_* from a factor measured in [43]. The second factor is the reciprocal density of *oriC* per unit *OD_450_* taken to be constant with growth rate at value 10^−9^ [33]. The third factor is in units of dosage ratio of an operon at location *m* to *oriC* given by Equation 7 with *C_μ_* = (220/3) − (40/3)*μ* as above. Promoter velocities 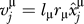 are therefore in units of initiations per minute per copy. Values in Table 5 are shown at only two significant figures to emphasize their highly approximate nature, owing to the approximately 10% uncertainty in the isoacceptor concentration measurements from which they are derived, and to the rough nature of the assumptions that went into the least squares estimation, and are probably underestimates for reasons discussed in the text. Figure 6 shows ratios 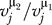 of promoter velocities at a high growth rate *μ_2_* and a lower growth rate *μ_1_*.

Circular statistical calculations were calculated in R with the additional CircStats package (S-plus original by Ulric Lund, R port by Claudio Agostinelli, available at http://cran.r-project.org/). To compare the number of tDNAs in leading and lagging strand operons by a permutation test, we calculated the sizes of the operons, where the size *s_j_* of the jth operon, 1 ≤ *j* ≤ *P* is 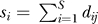 . The means and variances were similar among the two groups, with the leading group mean at 2.077 and the lagging group mean at 1.833, and the variances 2.474 and 2.5, respectively. We then carried out a permutation test sampling *R =* 10,000 permuted assignments of sizes to the leading and lagging strand groups using the standard equal-variance two-sample *t*-test as a test statistic and report the proportion 
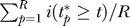
 ***,*** where *i* is an indicator function equal to one if its argument is true and zero otherwise, *t* is the value of the test statistic for the observed groups and 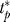 is the value of the test statistic for the *p*th permuted group assignment.

## Supporting Information

Dataset S1 Dong et al.'s tRNA Concentration Data with Classification of Isoacceptors This dataset contains Dong et al's Table 5 with concentration data, and the assignment of isoacceptor types to tRNAs: “major”, “minor,” and “neither.”
Units of concentration are uM. To reproduce the results of Figure 2 and the statistical two-sample tests, values for Pro1 and Pro3 should be added together, as should Thr1 and Thr3.
(3 KB TXT)

Dataset S2 Design Matrix for Least Squares Regression with 44 Operons and 11 Constraints This is the “correct” matrix used in the analysis, joining ribosomal operons* rrnC, rrnD,* and* rrnH*. Can be input directly into R.
(6 KB TXT).

Dataset S3 Concentration and Constraint Matrix to be Used with S2 (1 KB TXT).

Dataset S4 Automated Design Matrix for Least Squares Regression with 47 Operons and 13 Constraints Corresponds to tDNA clusters found with a clustering radius of 300 bp used in the paper, which splits apart ribosomal operons *rrnC, rrnD,* and* rrnH*. Can be input directly into R.
(6 KB TXT).

Dataset S5 Concentration and Constraint Matrix to be Used with S4 (1 KB TXT).

Dataset S6 OSCs and Other Correlates This table gives OSCs made with the “correct” design matrix Dataset S2 called “design_matrix.correct” and estimated by least squares regression through the origin. Additional operon properties are collected here.
(5 KB TXT).

Dataset S7 Design Matrix for Least Squares Regression with 43 Operons and 10 Constraints This is the same as the “correct” matrix but excludes the operon *thrX* discussed in the text. Can be input directly into R.
(7 KB TXT).

Dataset S8 Concentration and Constraint Matrix to be Used with Dataset S7
(1 KB TXT).

Dataset S9 Minimal Design Matrix for Least Squares Regression with 44 Operons and 10 Constraints This imposes minimal possible constraints on expression equality among ribosomal operons* rrnC, rrnD,* and* rrnH*. Can be input directly into R.
(5 KB TXT).

Dataset S10 Concentration and Constraint Matrix to be Used with Dataset S9
(1 KB TXT).

Figure S1 Log-Likelihood Profile for Box-Cox Test (4 KB EPS).

### Accession Numbers

The GenBank (http://www.ncbi.nlm.nih.gov/Genbank) accession number for the *E. coli* K12 MG1655 genome is NC_000913.

## Abbreviations

bp: base pair
OSC: operon standardized concentration
rDNA: ribosomal DNA
rRNA: ribosomal RNA
